# Epigenetic and Transcriptional Regulation of Spontaneous and Sensory Activity Dependent Programs During Neuronal Circuit Development

**DOI:** 10.3389/fncir.2022.911023

**Published:** 2022-05-18

**Authors:** Gabriele M. Pumo, Taro Kitazawa, Filippo M. Rijli

**Affiliations:** ^1^Laboratory of Neurodevelopmental Epigenetics, Friedrich Miescher Institute for Biomedical Research, Basel, Switzerland; ^2^Department Biozentrum, University of Basel, Basel, Switzerland

**Keywords:** spontaneous activity, sensory experience, sensory maps, transcription, epigenetics, chromatin, activity-regulated genes, immediate early genes

## Abstract

Spontaneous activity generated before the onset of sensory transduction has a key role in wiring developing sensory circuits. From axonal targeting, to synapse formation and elimination, to the balanced integration of neurons into developing circuits, this type of activity is implicated in a variety of cellular processes. However, little is known about its molecular mechanisms of action, especially at the level of genome regulation. Conversely, sensory experience-dependent activity implements well-characterized transcriptional and epigenetic chromatin programs that underlie heterogeneous but specific genomic responses that shape both postnatal circuit development and neuroplasticity in the adult. In this review, we focus on our knowledge of the developmental processes regulated by spontaneous activity and the underlying transcriptional mechanisms. We also review novel findings on how chromatin regulates the specificity and developmental induction of the experience-dependent program, and speculate their relevance for our understanding of how spontaneous activity may act at the genomic level to instruct circuit assembly and prepare developing neurons for sensory-dependent connectivity refinement and processing.

## Introduction

Throughout the development of sensory systems, the interplay between intrinsic developmental programs and neuronal activity-dependent mechanisms has been shown to orchestrate various steps of circuit wiring – from initial specification, migration, and axonal pathfinding decisions all the way to synaptic development and plasticity (Hensch, 2005; Spitzer, 2006; West and Greenberg, 2011). In particular, two types of neuronal activity – sensory-evoked activity and patterned spontaneous activity – have been implicated in instructing these processes.

Classic experiments have revealed that during defined postnatal critical periods of plasticity, inputs from the senses are key for the final precision of circuit wiring in the corresponding brain areas (Wiesel and Hubel, 1963; Hensch, 2005; Jabaudon, 2017; Reha et al., 2020). While interactions with the environment play key roles in brain region assembly and neuronal specification, developing circuits have been shown to possess robust and pervasive patterns of spontaneously generated activity during pre-sensory stages (Galli and Maffei, 1988; Blankenship and Feller, 2010; Martini et al., 2021). The physiological properties and functional roles of this type of activity are especially well described in developing sensory systems, where it is generally thought to instruct initial circuit wiring and prepare the brain for sensory processing. Indeed, many features of sensory maps – such as the topographic mapping of sensory inputs – are established with a significant degree of precision before sensory transduction even starts (Arroyo and Feller, 2016; Thompson et al., 2017; Kitazawa and Rijli, 2018; Antón-bolaños et al., 2019).

Activity-dependent gene regulation has emerged as a powerful mechanism to explain how activity is implemented at the molecular level to generate functional outputs relevant for neuronal connectivity and synaptic plasticity. Dedicated transcriptional and epigenetic chromatin programs are activated downstream of sensory activity and involve activity-dependent immediate early genes (IEGs) mainly encoding for transcription factors (TFs), which in turn regulate molecular effectors of circuit connectivity refinement and synaptic plasticity (Yap and Greenberg, 2018). The advent of modern genomic technologies has allowed to characterize this sensory program with unprecedented scope and detail, and has started to reveal how these genomic responses are tuned both to the cell type and the features of the stimuli that engage them (Gray and Spiegel, 2019; Tyssowski and Gray, 2019).

Similarly, progress in the imaging, recording, and manipulation of neuronal activity has led to a deeper understanding of the physiological patterns (Babola et al., 2018; Mizuno et al., 2018; Gribizis et al., 2019) and functional roles of spontaneous activity (Niculescu et al., 2018; Antón-bolaños et al., 2019; Nakashima et al., 2019; Ge et al., 2021; Tiriac et al., 2022), but little is known about the molecular mechanisms it deploys, especially at the level of chromatin and transcription. Individual effectors are starting to be identified (Hanson and Landmesser, 2004; Mire et al., 2012; Moreno-Juan et al., 2017; Nakashima et al., 2019), but a comprehensive knowledge of the dedicated chromatin and transcriptional programs involved in the response to spontaneous activity – including its similarities and distinct features compared to the sensory activity program – is currently missing.

Here, we review recent findings in activity-dependent transcriptional and epigenetic chromatin mechanisms, focusing also on work elucidating molecular targets of spontaneous activity and their functional roles in developing sensory systems. Furthermore, we review the emerging body of literature investigating the chromatin mechanisms regulating stimulus- and cell type-specific coupling between activity and transcription, and the developmental induction of the experience-dependent program. Based on these observations, we further speculate that sensory and spontaneous activity might work *via* distinct molecular programs. While such questions remain to be addressed by the field, this review aims to set a framework for the understanding of how different modalities of neuronal activity may control distinct molecular substrates for sensory circuit assembly.

## Spontaneous Activity in the Assembly of Sensory Circuits

Developmental neuroscientists have traditionally regarded circuit assembly as characterized by an early period during which intrinsic genetic mechanisms instruct broad connectivity, and a later period when neuronal activity refines it. Such a view has been challenged by findings showing that spontaneously-generated neuronal activity could be recorded arising from the sensory periphery before the start of sensory experience. Such recordings of spontaneous activity were carried out in the retina (Galli and Maffei, 1988; Wong et al., 1993), in the auditory nerve and brainstem (Lippe, 1994) and sensorimotor system (Landmesser and O’Donovan, 1984). Currently, spontaneous activity is regarded as a hallmark of developing circuits, and has been investigated in the visual, auditory, somatosensory and olfactory systems (Kirkby et al., 2013; Martini et al., 2021; Redolfi and Lodovichi, 2021). The circuits in these distinct sensory modalities have distinct patterns of generation, frequency and propagation of spontaneous activity, which are likely tailored to perform circuit-specific functions. Nevertheless, several common features have been proposed (reviewed in Blankenship and Feller, 2010). These include the presence of cells that act as pacemakers to produce specific patterns of activity, the robustness of spontaneous activity, and the role for transient, immature circuit features (e.g., transient connectivity or electrophysiological properties) that are key for the generation and propagation of patterned spontaneous activity.

Furthermore, electrical properties can also influence neuronal progenitor dynamics. Radial glia cells in the embryonic cortex exhibit calcium waves that have been implicated in the control of proliferation (Weissman et al., 2004), whereas the developmental progression of cortical neuron specification has been found to depend on the dynamics of the membrane potential in the progenitors (Vitali et al., 2018). Intrinsic programs can further determine cell receptiveness to extrinsic signals, in a process where these two parameters can reciprocally influence each other.

Overall, intrinsic spontaneous activity is now widely appreciated to be a key mechanism that predates extrinsic sensory inputs to instruct early steps of sensory circuit assembly (Hooks and Chen, 2006; Kirkby et al., 2013; Thompson et al., 2017). The patterns and function of spontaneous activity have been thoroughly reviewed (Arroyo and Feller, 2016; Leighton and Lohmann, 2016; Martini et al., 2021; Nakazawa and Iwasato, 2021). Here, we provide an overview of spontaneous activity in sensory systems, which is propaedeutic for the understanding of the molecular mechanisms downstream of spontaneous activity, discussed in the next chapter.

### Visual and Auditory Systems

Spontaneous activity is best understood in the visual and auditory systems, where it presents similarities in how it is generated, propagated and functionally implicated in circuit development. In both systems, spontaneous activity is generated by the immature sensory periphery of the mouse in a period which spans late embryonic stages to the start of sensory transduction (i.e., the opening of the external auditory canal and the eyes at around postnatal days 12–14).

In the retina, spontaneous activities in the form of wave-like patterns (i.e., retinal waves) are generated by different interneuron cell types with distinct circuit mechanisms throughout development, propagate through the retina, and ultimately activate retinal ganglion cells (RGCs; Blankenship and Feller, 2010). Type I waves are present at perinatal stages and depend on cholinergic signaling and gap junction (Bansal et al., 2000). Type II waves are cholinergic and cover the first postnatal week (Feller et al., 1996). Type III waves are glutamatergic and are present from the end of the previous phase to eye opening (Kerschensteiner, 2016). This diversity in the mechanisms of generation is also reflected by the changing physiological properties of the waves, including their frequency and size (i.e., the amount of cells that are activated by a wave; Maccione et al., 2014), which are key determinants of their function.

In the auditory system, spontaneous activity is generated in the inner ear by the cochlea (Tritsch et al., 2007; Wang and Bergles, 2015). There, glia-like cells called inner supporting cells (ISCs) as well as inner hair cells (IHCs, the mechanoreceptors for sound) are involved in a complex mechanism of ATP release by ISCs, indirect depolarization of IHCs and calcium spikes within IHCs which activate the glutamatergic excitation of spiral ganglion neurons (SGNs; Tritsch and Bergles, 2010; Tritsch et al., 2010a,b; Babola et al., 2021). This cascade of events leads to the spontaneous, correlated activation of neighboring clusters of SGNs throughout the entire period of pre-hearing development, and the frequency of activation is regulated by transient cholinergic innervation from the brainstem (Johnson et al., 2013; Clause et al., 2014; Babola et al., 2021).

Importantly, the spontaneous activity generated by the auditory and visual periphery is transmitted throughout the respective central pathways to activate relay stations in the hindbrain (only auditory), midbrain (inferior and superior colliculus), auditory and visual thalamus and cortex (Mooney et al., 1996; Hanganu et al., 2006; Ackman et al., 2012; Babola et al., 2018). Remarkably, the spatial and temporal features of correlated activity are preserved in these brain areas. In the visual system, spontaneous activity coming from the retina causes synchronized waves with similar spatiotemporal features spreading throughout the superior colliculus and in the primary visual cortex (V1; Ackman et al., 2012). In addition, in visual cortex, there are late activity patterns with distinct synchronization features that are not dependent on retinal waves, and might be generated within the cortex itself (Siegel et al., 2012; Gribizis et al., 2019; Wosniack et al., 2021). In the auditory system, spontaneous activity coming from the cochlea and transmitted by the brainstem activates the inferior colliculus and primary auditory cortex (A1) in highly synchronous patterns (Babola et al., 2018).

**FIGURE 1:**
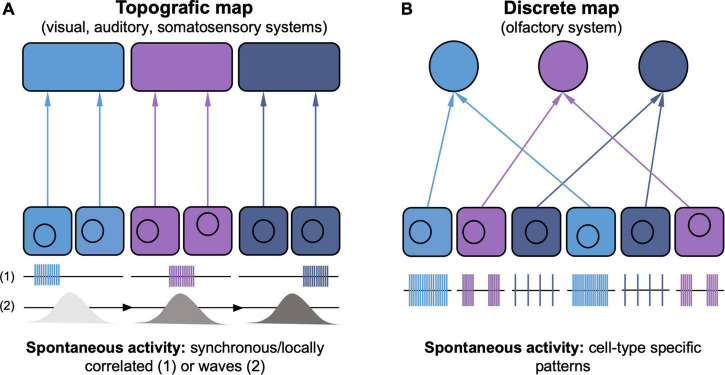
Distinct types of spontaneous activity instruct topographic *vs.* discrete sensory map development. **(A)** In the visual, auditory and somatosensory systems, topographic maps develop because spatial segregation of peripheral inputs is maintained throughout the central pathways. Spontaneous activity appears either as spatially confined bursts (1) or propagating waves (2), synchronizing neighboring neurons (indicated by matching color code). Spontaneous activity propagates from presynaptic to postsynaptic neurons maintaining its spatio-temporal features (not shown), thus supporting the topographic wiring logic by correlating topographically-matched neighboring neurons. **(B)** The discrete glomerulus map in the olfactory system maintains no information about the spatial arrangement of olfactory neurons in the periphery. Instead, neurons expressing the same olfactory receptor (indicated by matching color code), target the same glomerulus. Olfactory receptors instruct cell type-specific patterns of spontaneous activity, which show no spatial synchronicity, in turn regulating the complement of axon sorting molecules that define precise glomerulus targeting.

Visual and auditory spontaneous activities display features that reflect the topographic organization of the underlying circuits (Figure 1A). Neurons that will later respond to and process similar sound frequencies are positioned in close physical proximity along the cochlea and the entire auditory pathway, creating a tonotopic organization (Kandler et al., 2009). Spontaneous activity relayed by neighboring cochlear neurons synchronizes the firing of neurons with similar tonotopic properties, which are activated in isofrequency bands across the corresponding brain regions (Babola et al., 2018). Similarly, the waves propagating through the visual system allow the synchronization of the activity of neighboring neurons (Ackman et al., 2012), which respond to visual stimuli in close proximity in visual space, creating a retinotopic map. Inhibitory mechanisms that restrict the number of neurons activated by a spontaneous activity event have been identified, underscoring the importance of maintaining local activity synchronization (Leighton et al., 2021; Maldonado et al., 2021). Additionally, the directionality of retinal wave propagation in the superior colliculus has been show to mimic the optic flow generated by forward motion, further highlighting that these patterns are key to prepare the system for sensory processing (Elstrott and Feller, 2010; Ge et al., 2021).

These spatiotemporal properties of retinal and cochlear patterned activities, and the observation that topographic features of auditory and visual maps are fairly precise even before sensory transduction, suggest a causal role for instructing patterned connectivity (Figure 1A). Indeed, functional experiments in the visual system have shown that blocking retinal waves leads to profound defects in visual map assembly (McLaughlin et al., 2003; Chandrasekaran et al., 2005; Torborg and Feller, 2005). More recently, approaches allowing the manipulation of individual features of retinal waves (e.g., their frequency, size or degree of correlation between the two eyes) have allowed to dissect their instructive role for retinotopy (Xu et al., 2011, 2015; Burbridge et al., 2014) and the segregation of the inputs from the two eyes (Zhang et al., 2012; Burbridge et al., 2014; Xu et al., 2015; Tiriac et al., 2018). The frequency of the waves specifically is key for input segregation, while the wave size instructs retinotopy. Instead, the directionality of wave propagation has been shown to instruct the formation of firing properties that relate to the direction of the visual stimulus (i.e., direction selectivity; Ge et al., 2021; Tiriac et al., 2022). In the auditory system, the tonotopic refinement in the brainstem is compromised when the frequency of cochlear activity is altered (Clause et al., 2014, 2017).

Collectively, these results show that patterned spontaneous activity in visual and auditory systems before sensory experience has specific features that have key roles in instructing the development of the respective sensory maps (Figure 1A).

### Somatosensory System

In mice, the somatosensory pathway – i.e., the whisker-to-barrel cortex pathway, in the scope of this review – is already active at birth, almost two weeks before eyes and ears open (Antón-bolaños et al., 2019; Cai et al., 2021). Whisker stimulations at perinatal stages (E18.5-P0) are relayed by the trigeminal ganglion, the trigeminal column in the brainstem and through the lemniscal pathway to ventral posterior medial (VPM) thalamus and primary somatosensory cortex (S1; Kitazawa and Rijli, 2018). In the newborn mouse, passive sensation arising from interactions with objects in the cage, littermates and the mother cause cortical activation (Akhmetshina et al., 2016), well before the onset of active whisking and exploratory behavior in the second postnatal week (Iwasato and Erzurumlu, 2018; Yang et al., 2018). Consistent with this precocious maturation, the somatotopic organization of neurons into barrelette, barreloid and barrels from brainstem to thalamus to cortex, respectively, is already present at P4, and depends on sensory activity from the whiskers (Erzurumlu and Gaspar, 2012).

This early form of sensory stimulation elicits distinct patterns of cortical activation (reviewed in Luhmann and Khazipov, 2018). Spindle bursts and gamma oscillations are locally restricted to individual cortical columns, the early templates for barrels, and correlated between thalamus and cortex (Figure 1A), while long oscillations are less frequent but correlate larger networks of cortical neurons (Yang et al., 2009, 2013; Minlebaev et al., 2011). Additionally, the whiskers of newborn rodents twitch spontaneously during sleep, and sensory feedback from such movements (i.e., reafferentation) can also drive cortical activation in the form of spindle bursts (Khazipov et al., 2004; Tiriac et al., 2012, 2014; Dooley et al., 2020).

The presence of such spontaneous sensation and self-generated movement complicates the interpretation of early postnatal activity patterns, but spontaneous activity likely independent of these processes has been recently reported (Nakazawa and Iwasato, 2021). A “patchwork-type” pattern of spontaneous activity, where both the neurons belonging to the same barrel as well as the thalamocortical axons (TCAs) projecting from a barreloid fire synchronously, and different barrels fire in a successive manner, was described *in vivo* in S1 from P0 to P5 (Mizuno et al., 2018, 2021; Nakazawa et al., 2020). The origin of this type of activity is still unclear, but its spatiotemporal properties and its occurrence during the period of thalamocortical refinement seem to be uniquely poised to instruct the somatotopic development of the barrel map. Spindle bursts and gamma oscillations have also been shown to regulate thalamocortical topography (Luhmann and Khazipov, 2018) and cortical cell death (Molnár et al., 2020), reviewed in the next chapter. However, precise manipulations that allow to disentangle the contribution of co-occurring sensory-evoked and spontaneous activity are needed to better understand its functional role.

In the somatosensory system, prenatal thalamic spontaneous activity has been described in *ex vivo* slices (reviewed in Martini et al., 2021). Initially present as uncorrelated calcium transients, from mid-gestation until birth the somatosensory, visual and auditory thalamic nuclei produce waves of spontaneous activity that spread between the different sensory modalities by gap junctions (Moreno-Juan et al., 2017). These waves are also relayed to the cortex and control the area size of the different sensory modalities by regulating TCA branching (Moreno-Juan et al., 2017). At perinatal stages, localized whisker pad or thalamic stimulations elicit localized cortical responses, suggesting the present of a barrel “protomap.” In mice where these waves are specifically suppressed, such local responses activate larger cortical areas (Antón-bolaños et al., 2019), implicating spontaneous thalamic activity in the creation of functional cortical maps (Figure 1A) that are then further refined by patchwork-type and sensory activity patterns after birth (Antón-bolaños et al., 2019; Nakazawa and Iwasato, 2021).

### Olfactory System

The olfactory system presents a functional organization that is distinct from the somatotopic, retinotopic and tonotopic arrangements observed in the sensory modalities described above (Figure 1B). Indeed, there is no apparent spatial organization of olfactory sensory neurons in the olfactory epithelium to represent features of the sensory stimulus. Instead, each olfactory neuron expresses only one type of olfactory receptor. Olfactory neurons expressing the same olfactory receptor then converge onto the same location of the olfactory bulb and synapse with second order neurons, forming a glomerulus (Nishizumi and Sakano, 2015).

The targeting of olfactory sensory neurons to the appropriate glomerulus is highly regulated by guidance molecules and signaling downstream of the olfactory receptor, but in the absence of spontaneous activity the map results unrefined and several axons target the wrong glomerulus (Yu et al., 2004; Lorenzon et al., 2015). While spontaneous activity is necessary for map refinement, manipulations that affect odor-evoked activity have no effect on this process, in contrast to the role of experience in the other sensory modalities (Lin et al., 2000; Redolfi and Lodovichi, 2021).

The differences in the organization of the olfactory system makes it difficult to explain the role of spontaneous activity by using mechanisms emerging from other sensory systems. In the auditory, somatosensory and visual systems, spontaneous activity that locally correlates neurons with similar topographic properties is thought to be key in mediating Hebbian plasticity rules, whereby neurons with correlated activity preferentially wire together (Figure 1A; Leighton and Lohmann, 2016; Nakashima et al., 2021). In the olfactory system such a wiring logic would not work, as the spatial proximity of olfactory sensory neurons is not instructive for their wiring and neighboring neurons show diverse and uncorrelated activity patterns (Figure 1B; Connelly et al., 2013). Recent work has shown that the olfactory receptor has a fundamental role in determining the spontaneous activity patterns of individual neurons. Neurons with the same olfactory receptor, which target the same glomerulus (Figure 1B, neurons of same color) show similar patterns of activity; switching the type of receptor also switches the type of activity, and inducing a different activity pattern changes glomerular targeting (Nakashima et al., 2019). These results elegantly highlight that spontaneous activity acts cell-autonomously to establish the targeting properties of individual olfactory neurons and support the distinct logic of the wiring of the olfactory system (Figure 1B; Nakashima et al., 2021).

## Cellular Functions and Transcriptional Targets Downstream of Spontaneous Activity

The body of work presented in the previous chapter makes a strong argument for the role of patterned, developmentally regulated, sensory modality-specific spontaneous activity in the development of sensory systems. However, much less is known about the molecular effectors acting downstream of spontaneous activity (see Yamamoto and López-Bendito, 2012 for an earlier review on the topic focusing on axon guidance). For example, neurotransmitter specification in *Xenopus* has been shown to depend on spontaneous activity (Demarque and Spitzer, 2010; Marek et al., 2010; reviewed in Spitzer, 2012). Early forms of neuronal excitability, such as calcium transients, and the processes they regulate have also been thoroughly reviewed (Spitzer, 2006; Rosenberg and Spitzer, 2011). In this chapter, we describe the knowledge on the cellular processes controlled by spontaneous activity and the molecular effectors through which they are regulated during the development of sensory systems, which are summarized in Table 1.

**TABLE 1:** List of molecular effectors regulated by spontaneous neuronal activity during sensory circuit development described in this review. Their molecular role, the develpmental processes they regulate, the type of activity that induces them and the corresponding references are indicated.

Gene	Molecular function	Developmental process	Type of activity	References
**Axonal growth, guidance and branching**				
EphA4	Axon guidance receptor	Spinal motoneuron pathfinding	Spontaneous activity waves in embryonic spinal cord	Hanson and Landmesser, 2004; Kastanenka and Landmesser, 2010
EphB1				

Kirrel2				
PCDH10	Axon guidance, adhesion and fasciculation molecules	Segregation of olfactory neuron axons targeting olfactory bulb	Olfactory receptor –specific patterned spontaneous activity	Nakashima et al., 2019
Sema7A				

Robo1	Axon guidance receptor – regulator of axon elongation speed	Thalamocortical axon pathfinding	Prenatal thalamic spontaneous activity	Mire et al., 2012
Dcc				Castillo-Paterna et al., 2015

Rorβ	Nuclear receptor – regulator of axon branching	TCA branching in somatosensory cortex	Prenatal thalamic waves	Moreno-Juan et al., 2017

**Migration and circuit integration of interneurons**				
Dlx1	Transcription factor	Migration and layer-specific allocation of cortical interneurons	Not determined: Spontaneous activity or early sensory activity during first postnatal week	De Marco García et al., 2011
ELMO1	Regulator of cell motility			
Satb1	Chromatin factor	Maturation and circuit integration of cortical interneurons		Denaxa et al., 2012; Close et al., 2012

**Synaptic plasticity and refinement**				
H2-D^b^, H2-K^b^ (MHC class I)	In neurons: signaling regulating synaptic pruning	Synaptic plasticity/ elimination for eye-specific segregation at retino-thalamic synapse	Retinal waves	Corriveau et al., 1998; Lee et al., 2014

proBdnf, Bdnf	Secreted neurotrofic factors	Clustering and consolidation of synchronously active synapses in V1	Cortical or retinal spontaneous activity	Winnubst et al., 2015; Niculescu et al., 2018

### Axon Guidance and Neuronal Migration

Axon guidance is a highly regulated process (Stoeckli, 2018). While here we report examples where axonal targeting is regulated by activity (Table 1, top), there are also instances where this process is controlled by activity-independent hard-wired molecular mechanisms. Genetic control of guidance mechanisms and activity-dependent refinement might complement each other while keeping distinct and independent roles, as is thought to be the case for the formation of retinotopic maps in the visual system (Cang et al., 2005, 2008). While retinotopy depends both on Eph/Ephrin gradients and spontaneous activity, Eph/Ephrin levels are not regulated by activity and initial axon targeting is also independent of spontaneous activity (Benjumeda et al., 2013). Ultimately, the extent to which activity and genetics interact or complement each other by acting sequentially is likely highly system-specific.

Early studies in the field have shown that spontaneous activity in the form of calcium transients can influence various aspects of axon growth and navigation in spinal neurons of *Xenopus* tadpoles (Gu and Spitzer, 1995; Gomez and Spitzer, 1999; Spitzer, 2006). In chick embryo spinal cord, pharmacological inhibition of spontaneous activity was shown to result in guidance defects of motoneurons innervating the limbs, potentially through the regulation of guidance molecule EphA4 (Hanson and Landmesser, 2004). Optogenetically restoring normal activity patterns prevented the formation of these defects and restored normal levels of EphA4 (Kastanenka and Landmesser, 2010).

In the mouse olfactory system, axonal adhesion proteins contribute to sort sensory neuron axons, so that axons from neurons expressing the same olfactory receptor converge to the appropriate glomerulus (Nishizumi and Sakano, 2015). Neurons expressing the same olfactory receptor produce similar spontaneous activity patterns, while activity patterns are remarkably different across neurons (Nakashima et al., 2019). Additionally, olfactory receptor expression and activity type also correlate with the complement of adhesive molecules expressed by the neurons (e.g., Kirrel2, PCDH10, Sema7A). By genetic manipulations that substitute the olfactory receptor and optogenetic manipulations to simulate a different pattern of activity, a direct link between the pattern of activity, the complement of expressed adhesion molecules, and the targeting of the glomerulus was established (Nakashima et al., 2019).

In the mouse thalamocortical system, prenatal spontaneous thalamic activity (Moreno-Juan et al., 2017) has been shown to regulate two aspects of TCA targeting: the speed of axon elongation and the branching of axonal terminals within cortex. Both spontaneous thalamic activity and TCA speed are developmentally regulated, so that the frequency of spontaneous bursts and TCA speed decrease as the axons enter the cortex (Mire et al., 2012). The guidance receptors Robo1 and Dcc are expressed in a spontaneous activity-dependent manner and control TCA speed by acting in opposite ways, slowing down and accelerating elongation rate, respectively (Mire et al., 2012; Castillo-Paterna et al., 2015). Additionally, *in vitro* reporter assays suggest that NF-κB and AP1 (i.e., the TF complex formed by IEGs Fos and Jun) binding sites in the regulatory regions of Robo1 and Dcc, respectively, control their activity-dependent expression. However, whether NF-κB, Jun and Fos are induced by thalamic activity and actually bind these sites *in vivo* remains to be investigated. While thalamic activity regulates targeting speed in a cell-autonomous manner, blocking of calcium waves in the prenatal visual thalamus has been reported to delay its innervation by cortico-thalamic axons, suggesting a non cell-autonomous timing mechanism (Moreno-Juan et al., 2020).

Within cortex, TCA axonal terminals branch to innervate the correct cortical territory and these branching patterns are important for the formation of topographic maps. *In vitro* experiments using thalamocortical slices in culture demonstrated that both pre- and post-synaptic activity is necessary for correct branching of TCAs (Uesaka et al., 2005, 2007; Yamada et al., 2010; Matsumoto et al., 2016). Additionally, molecular effectors such as Netrin4 and HDAC9, an epigenetic regulator that represses activity-dependent gene expression mediated by Mef2, have been shown to regulate branching in these cultures, but whether sensory or spontaneous activity regulate their function *in vivo* remains to be determined (Sugo et al., 2010; Hayano et al., 2014; Alchini et al., 2017). *In vivo* studies have shown that prenatal thalamic waves regulate the size of the primary sensory areas in the cortex (Moreno-Juan et al., 2017). This effect is mediated by the activity-dependent expression of *Rorβ*, which controls TCA branching. Manipulations that increase the frequency of waves in the somatosensory thalamus lead to increased *Rorβ* expression, increase in TCA branching and enlargement of S1 size (Moreno-Juan et al., 2017). However, the gene regulatory program that connects thalamic waves to the expression of this regulator of dendritic branching has not been explored.

Spontaneous cortical activity is also key for the formation of inter-hemispheric projections of callosal neurons in V1 and S1 (Mizuno et al., 2007, 2010; Wang et al., 2007; Rodríguez-Tornos et al., 2016). Both spatial features and the developmental timing of spontaneous activity are instrumental for the targeting of these commissural axons (Suárez et al., 2014; Tezuka et al., 2021). Additionally, distinct patterns of network activity are present in cortical populations during these developmental stages (Siegel et al., 2012), but they might be differentially required for callosal projections (Tezuka et al., 2021). Activity-regulated molecular targets that instruct callosal projections, and their induction by distinct patterns of spontaneous activity remain to be characterized.

Apart from axon guidance, neuronal migration is another early aspect of circuit formation which has been shown to be dependent on activity. Early studies have shown how different properties of spontaneous calcium transients regulate migration of granule cells in the cerebellar cortex (Komuro and Rakic, 1992, 1996; Komuro et al., 2015). In the developing neocortex, post-migratory projection neurons exhibit fewer calcium transients than migratory neurons, and increasing activity leads to a premature stop in migration and permanent laminar mispositioning (Bando et al., 2016; Hurni et al., 2017). Transcriptomic analysis revealed that genes upregulated by increased activity were part of the program related to morphogenesis and cytoskeletal remodeling, fitting with the observation that upon stopping migration these neurons prematurely undergo neurite outgrowth in the wrong cortical layer, but do not acquire the cognate layer identity (Hurni et al., 2017).

### Migration and Circuit Integration of Interneurons

Similarly to excitatory neurons, cortical GABAergic interneurons also require neuronal activity, potentially including spontaneous activity, for early developmental processes, initially thought to be activity-independent (Babij and De Marco Garcia, 2016; Wamsley and Fishell, 2017). Blocking neuronal activity by hyperpolarization was shown to influence migration, cortical layer allocation, dendritogenesis and axonogenesis of selected subtypes of GABAergic interneurons during specific time windows of postnatal development (De Marco García et al., 2011). The effect of activity on migration was found to be dependent on a transcriptional program involving the activity-dependent transcription factor (TF) Dlx1 and its regulation of *ELMO1*, a gene involved in cytoskeletal rearrangement. Interestingly, ELMO1 is only expressed by the interneuron subclasses that depend on activity for correct migration, indicating the presence of a cell type-specific activity-dependent program regulating migration (De Marco García et al., 2011).

The transcription factor Satb1 was also shown to be a potential activity-dependent factor for the development of specific classes of GABAergic interneurons (Denaxa et al., 2012). Removal of *Satb1* leads to reduced inputs onto these interneurons, their death during early postnatal development, and reduced functional inhibition of cortical circuits (Close et al., 2012). As activity was found to be required for these processes in distinct time windows during the first postnatal weeks, it is not possible to exclude contributions from early sensory-evoked activity. Indeed, the axo-dendritic development and circuit integration of layer I interneurons in the somatosensory cortex was shown to depend on sensory inputs relayed by the thalamus (De Marco García et al., 2015), and in turn these inhibitory neurons ensure topographic mapping in S1 by controlling barrel map size and the number of excitatory neurons recruited by whisker stimulations (Che et al., 2018). Nevertheless, interneurons in somatosensory cortex have been recently shown to exhibit spontaneous activity patterns similar to those found in cortical excitatory neurons (Mizuno et al., 2018), and to be key regulators of early cortical spontaneous activity, so it is plausible that early activity might also regulate aspects of their migration, connectivity, and circuit integration (Babij and De Marco Garcia, 2016; Che et al., 2018; Modol et al., 2020).

### Synaptic Plasticity and Refinement

While the evidence discussed above highlights how spontaneous activity can regulate molecular mechanisms for migration and axonal targeting before synapse formation, several functional studies have proposed that distinct features of spontaneous activity are important in shaping synaptic connectivity during the refinement of sensory maps (Arroyo and Feller, 2016). The formation of retinotopy and eye-specific segregation in the retinothalamic and retinocollicular maps have been especially suited to study the contribution of both spontaneous and sensory-evoked activities in instructing these processes (Hooks and Chen, 2006; Thompson et al., 2016, 2017). Despite this, our knowledge of the molecular mechanisms engaged by spontaneous activity during synaptic refinement remains scarce, and the activity-dependent programs underlaying synaptic plasticity remain best characterized in the context of sensory experience (Louros et al., 2014; Cheadle et al., 2018, 2020; Yap and Greenberg, 2018).

The pruning of synapses is a key process occurring during the refinement of sensory maps. Eye-specific segregation of retinal input in the visual thalamus is a model system to study activity-dependent synapse elimination (Faust et al., 2021). Several molecules related to immune system signaling, such as MHC class I molecules, pentraxins and components of the complement pathway have been shown to be key determinants of synaptic elimination at the retinogeniculate synapse (Stevens et al., 2007; Schafer et al., 2012; Stephan et al., 2012). MHC class I genes were found to be regulated both by retinal waves and sensory experience at subsequent stages of retinogeniculate refinement (Corriveau et al., 1998). Mice lacking these molecules, as well as components of their receptor, or neuronal pentraxins, show impaired eye-specific segregation (Huh et al., 2000; Bjartmar et al., 2006) and defects in synaptic strength and elimination in cortex (Glynn et al., 2011; Adelson et al., 2016). Importantly, the deletion of two key components of MHC class I (H2-D^b^ and H2-K^b^) leads to defective synaptic elimination and eye-specific segregation in the presence of normal retinal waves, and these defects could be rescued by selectively overexpressing one of the MHC class I genes in neurons (Lee et al., 2014). The neuronal-specific function of these genes was further shown to involve the induction of long-term depression through the regulation of AMPA receptors, linking activity-dependent synaptic weakening to their eventual elimination (Lee et al., 2014). While this study reveals a major molecular pathway involved in spontaneous activity-dependent synaptic refinement, how MHC class I molecules are transcriptionally regulated by activity, as well as whether MHC signaling leads to transcription-dependent effects remains unexplored.

A different molecular mechanism for spontaneous activity-dependent synaptic plasticity was discovered in visual cortex. Stemming from the observation that synapses that are in close proximity along a dendrite are more likely to be active simultaneously (Kleindienst et al., 2011), two studies went on to show that a plasticity mechanism involving Bdnf consolidates synapses that fire synchronously while depressing synapses that fire asynchronously (Winnubst et al., 2015; Niculescu et al., 2018). Specifically, this push and pull model suggests that synchronously active synapses become clustered through the conversion of secreted pro-Bdnf into Bdnf, and signaling through TrkB receptors. Asynchronous synapses instead fail to convert pro-Bdnf into Bdnf and thus pro-Bdnf induces synaptic depression by signaling through the p75^NTR^ receptor (Niculescu et al., 2018). Such a mechanism might be suitable to explain how spontaneous activity favors the connectivity of synchronized neurons. However, a comprehensive understanding of the transcriptional programs that support spontaneous activity-dependent plasticity is lacking.

### Cell Death

Apoptosis is a key event that shapes developing neuronal circuits, and has been studied extensively in the sensory systems, where it co-occurs with activity-dependent circuit development. Early studies using cortical cultures showed that cell survival can be increased by the activation of voltage-dependent calcium channels, which leads to the expression of neurotrophic factor Bdnf. These results suggest that activity is a regulator of neuronal survival through the activity-dependent expression of neurotrophic factors (Ghosh et al., 1994). Further support for the role of activity-dependent transcription was given by findings that implicated NMDA receptor signaling in activating distinct transcriptional programs that selectively promote either neuronal death or survival. This also suggests that neuronal activity *in vivo* could cause both outcomes – survival and apoptosis – and that the engagement of transcriptional programs is key to cause a specific outcome (Zhang et al., 2007).

Work combining *in vivo* observations and *in vitro* organotypic cultures showed that neuronal apoptosis occurs in the neonatal somatosensory cortex, and that patterned spontaneous activity similar to the one observed *in vivo* promotes neuronal survival through the activation of NMDA receptors, voltage-dependent calcium channels, CREB and neurotrophin receptors (Heck et al., 2008). Importantly, disrupting the pattern of the spontaneous activity leads to an increase in apoptosis, while stimulating neurons with physiological spindle bursts and gamma oscillations leads to increased survival, suggesting that features of the stimulation, and not just activity *per se*, might instruct neuronal survival (Heck et al., 2008; Golbs et al., 2011) by regulating the levels of activity-regulated genes (ARGs) and apoptotic factors (Wong Fong Sang et al., 2021). Additionally, differences in the levels of activity across developing cortical regions control area-specific levels of apoptosis (Blanquie et al., 2017).

Recent evidence has also shown that the activity-dependent elimination of cortical GABAergic interneurons – a class of neurons that shows high levels of apoptosis during circuit integration (Southwell et al., 2012) – is an important event in generating functional sensory circuits. Importantly, early network activity dynamics determine the fine-tuning of interneuron numbers, which has implications for the homeostasis (Denaxa et al., 2018) and functional topography of adult circuits (Duan et al., 2020). Activity-dependent interneuron elimination is subtype-specific and regulated by activity-dependent calcineurin signaling (Priya et al., 2018). A recent report directly linked spontaneous activity from two sources – retinal waves and spontaneous activity from callosal axons – and the apoptosis of interneurons in the binocular zone of the visual cortex before opening (Wang et al., 2021). The suppression of spontaneous activity from either source during the time-window of normal apoptosis leads to the ectopic survival of interneurons, which in turn affects the emergence of proper binocular visual properties. Early-postnatal activity also regulates the disappearance of transient neuronal populations such as Cajal-Retzius neurons, a key event for correct cortical wiring, but the nature of the activity remains to be investigated (Riva et al., 2019). While these studies suggest that neuronal survival and apoptosis might be dynamically regulated by spontaneous activity during sensory circuit development, the molecular mechanism that regulate death *vs.* survival, and their reliance on the activation of dedicated gene programs remains to be investigated.

Overall, the evidence presented in this chapter highlights how spontaneous activity acts throughout the development of sensory circuits, and is fundamental for processes taking place both before and after synapse formation. Many of these steps in circuit assembly rely on regulation of gene expression, and the molecular effectors of spontaneous activity are emerging. Additionally, upstream of spontaneous activity, transcription factors such as Cux1 and COUP-TFI/Nr2f1 regulate ion channel expression to precisely set the intrinsic firing properties of developing neurons (Rodríguez-Tornos et al., 2016; Del Pino et al., 2020). However, a detailed understanding of the epigenetic and transcriptional programs engaged by spontaneous activity, including the identity of TFs and their binding to activity-regulated enhancers, as well as the differences compared to the subsequent experience-dependent programs, is currently lacking.

## Transcriptional and Chromatin Regulation of Activity-Regulated Genes

Activity dependent-transcription drives the long-term adaptation of neurons to stimulations both during postnatal critical periods of sensory experience-dependent circuit development and in the adult during learning and memory formation (Yap and Greenberg, 2018). Neuronal depolarization caused by sensory experience activates synapse-to-nucleus calcium-dependent signaling pathways that post-translationally modify TFs to induce transcription of ARGs. Among ARGs, IEGs are rapidly induced without the need for protein synthesis and mainly code for TFs. IEG TFs then induce a second wave of late response genes (LRGs), which are the effectors of many neuronal processes revolving around synaptic plasticity and connectivity. Both the diversity and function of signaling pathways downstream of neuronal activity (Flavell and Greenberg, 2008; West and Greenberg, 2011) as well as the identity and functional roles of many IEGs and LRGs, have been reviewed thoroughly (Leslie and Nedivi, 2011; Benito and Barco, 2015; Yap and Greenberg, 2018). Here, we focus on recent advances in understanding the regulation of ARGs in the context of chromatin. Epigenetic chromatin mechanisms are emerging as important regulators of the developmental induction of ARGs as well as the heterogeneity and specificity of experience-dependent programs. After presenting these features, we speculate how these findings might also be of importance to understand genome regulation downstream of spontaneous activity.

### Chromatin Regulation of Immediate Early Genes

A hallmark feature of IEGs is their rapid stimulus-dependent transcriptional induction, which can happen in a matter of minutes (Greenberg and Ziff, 1984; Greenberg et al., 1986). This observation points towards the presence of regulatory mechanisms enabling rapid, but controlled induction at all levels of the signal transduction pathway (Flavell and Greenberg, 2008; West and Greenberg, 2011; Yap and Greenberg, 2018). Transcriptional and epigenetic regulation has emerged as a key mechanism in this regard. The promoter elements of ARGs such as *Bdnf* have complex architectures that allow fine tuning of their transcription (Flavell and Greenberg, 2008). More recent work has used genome-wide profiling of epigenetic marks and of transcriptional regulator binding to identify activity-dependent enhancers that are crucial for IEG expression (Kim et al., 2010; Malik et al., 2014). These studies have revealed that, in adult neurons, both promoters and enhancers of IEGs have distinct features that poise these genes for rapid activation. In unstimulated neurons, IEG (e.g., *Fos*) enhancers and promoters are accessible and marked by transcription permissive chromatin modifications such as H3K4me1 and H3K4me3, respectively (Kim et al., 2010; Tyssowski et al., 2018). These elements even carry modest basal levels of transcriptionally active H3K27ac mark in unstimulated neurons (Malik et al., 2014; Kitazawa et al., 2021). Regulatory elements are also pre-bound by transcriptional activators such as CREB and SRF, which are post-translationally activated in a stimulus-dependent manner downstream of calcium signaling (West and Greenberg, 2011). Additionally, RNA polymerase II (RNAPII) is stalled downstream of the transcriptional start site, so that transcriptional induction of IEGs requires RNAPII engaging in productive elongation (Saha et al., 2011). Importantly, this poised state is not found at delayed primary response genes (PRGs) nor at LRGs, underscoring its relevance for the fast induction kinetics of IEGs (Tullai et al., 2007; Saha et al., 2011; Tyssowski et al., 2018).

Upon neuronal activation and synapse-to-nucleus calcium signaling, CREB, SRF and other pre-bound constitutive TFs are phosphorylated and can recruit effectors that regulate transcriptional activation of IEGs (West and Greenberg, 2011; Yap and Greenberg, 2018). At promoters, phosphorylated CREB (CREB-P) recruits histone acetyltransferase CBP, increasing local histone acetylation. This in turn leads to recruitment of the positive transcription elongation factor p-TEFb, which leads to productive elongation of RNAPII via release of negative elongation factor (NELF) and phosphorylation in Ser2 of RNAPII C-terminal domain (Saha et al., 2011; Chen et al., 2018). At enhancers, calcium signaling recruits RNAPII, which leads to transcription of enhancer RNAs (Kim et al., 2010). These are important to release stalled RNAPII at the promoter and initiate productive transcription (Schaukowitch et al., 2014). CBP binding of enhancer RNAs induces deposition of H3K27ac (Bose et al., 2017), a signature of activity-induced enhancers (Malik et al., 2014). Additionally, it has been shown that H3K27 acetylation of enhancers and promoters of IEGs is relevant for the control of the frequency of transcriptional burst dynamics and thus the fine-tuning of expression levels (Chen et al., 2019).

### Developmental Immediate Early Gene Regulation Before Sensory Stimulation

**FIGURE 2:**
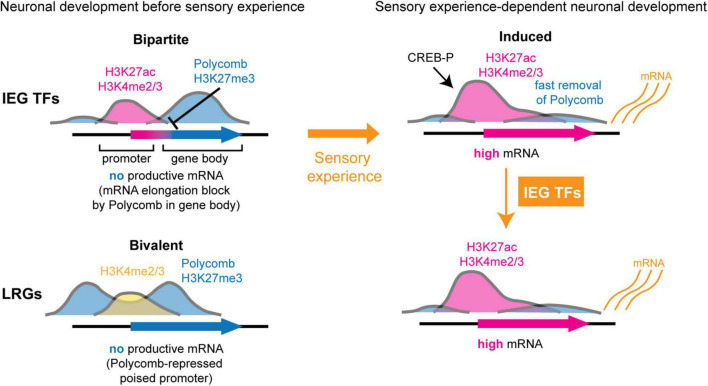
Polycomb-dependent regulation of activity response genes during neuronal development. At pre-sensory stages (left), immediate early genes (IEGs) and downstream LRGs display distinct chromatin profiles of Polycomb-dependent repression, preventing their precocious ectopic induction by environmental stimuli. LRGs carry bivalent promoters, which are simultaneously marked by permissively active H3K4methyl 2/3 (H3K4me2, H3K4me3) and Polycomb-repressive H3K27me3 histone modifications, and maintain a transcriptionally poised state with non productive transcription **(bottom left)**. In contrast, IEGs display a bipartite profile with H3K27acetyl (H3K27ac)+/H3K4me2/3+ active promoters but with Polycomb H3K27me3 on gene bodies **(top left)**. Bipartite IEGs carry active promoters initiating transcription; however, productive mRNA elongation is inhibited due to H3K27me3+ on gene bodies (inhibition sign). At the onset of sensory experience (right), sensory-driven neuronal activity induces phosphorylation of CREB (CREB-P) and resolves Polycomb-dependent gene body repression of IEGs, resulting in rapid productive transcriptional induction of IEGs **(top right)**. Transcription factors (TFs) encoded by IEGs in turn regulate downstream LRGs whose bivalent Polycomb-dependent chromatin signature is resolved from a transcriptionally poised in pre-sensory stages to an active state **(bottom right)**.

While the elucidation of chromatin mechanisms of IEG induction by sensory-evoked activity has seen much progress, our understanding of how IEGs are developmentally regulated before sensory stimulation is still poorly understood. Kitazawa and colleagues used comprehensive genome-wide approaches to analyze the chromatin state and regulation of ARGs during the development of whisker-related barrelette neurons in the brainstem (Kitazawa et al., 2021). This study found that during early development, prior to their sensory activity-dependent induction, IEGs are embedded in a specific Polycomb chromatin signature, named “bipartite” (Figure 2; Kitazawa et al., 2021). Polycomb chromatin factors are repressive regulators of gene expression during development (Schuettengruber et al., 2017). Bipartite genes have active promoters carrying the H3K27ac and H3K4methyl2/3 (H3K4me2, H3K4me3) histone marks; however, their gene bodies are marked by the Polycomb repressive histone modification H3K27me3 (Figure 2, top left). Polycomb marking on the gene body prevents stalled RNAPII from carrying out transcriptional elongation, resulting in IEG mRNA unproductive transcription. Upon stimulus-induced neuronal depolarization at perinatal stages, gene body H3K27me3 is fastly removed and RNAPII can productively transcribe bipartite genes. Interestingly, the bipartite chromatin state is also present in developing cortical neurons as well as in non-neuronal tissues. Bipartite genes (about 100–200 per cell type) are enriched in IEGs and in genes involved in calcium-regulated signaling pathways. The bipartite signature might thus be a common developmental mechanism that regulates the timing, rapidity, and amplitude of transcriptional activation of rapid stimulus response genes, including IEGs. Conversely, the majority of known LRG promoters display, at prenatal pre-sensory stages, a “bivalent” chromatin organization (Bernstein et al., 2006; Kitazawa et al., 2021), which is characterized by high levels of the Polycomb repressive H3K27me3 and active H3K4me2/3 histone modifications, respectively, maintaining genes in a repressed though transcriptionally poised state ready to be induced following IEG rapid induction (Figure 2, bottom).

In developing somatosensory barrelette neurons, IEGs are induced at perinatal stages, likely at the onset of sensory transduction from the whiskers. The Polycomb H3K27me3 mark is rapidly removed from gene bodies and the bipartite chromatin state is resolved in early postnatal neurons (Figure 2, top; Kitazawa et al., 2021). The role of Polycomb in ARG regulation at postnatal stages is still to be investigated. However, a recent study showed that in adult neurons activity-dependent *Bdnf* induction was regulated by Polycomb de-repression and CREB/CBP/JMJD3 activation in promoters (Palomer et al., 2016). Another developmental mechanism of IEG expression is set in place during early postnatal neuronal development, and involves DNA methylation of the gene body (Stroud et al., 2017). The DNA methyltransferase Dnmt3a deposits methylation at CA sites (mCA) across the genome, preferentially targeting the gene bodies of lowly expressed genes. Once deposited, mCA is bound by the methyl-DNA-binding factor MECP2, which negatively regulates gene expression. Inducing IEGs in a stimulus-dependent manner in early postnatal life results in lower Dnmt3a binding and lower mCA methylation in adult neurons. This mechanism might fine-tune the expression of ARGs during the neuronal postnatal life while still allowing for stimulation-dependent transcription. The successive temporal time windows in which the developmental Polycomb bipartite state and mCA deposition occur at IEG gene bodies, might indicate a sequential role for these epigenetic marking mechanisms to control the timing and extent of IEG transcriptional induction from development to adulthood.

### 3D Chromatin Organization

In addition to chromatin and DNA modifications, the 3D organization of chromatin is also emerging as a key regulator of activity-dependent gene expression (Dileep and Tsai, 2021). *Fos* and *Arc* induction was shown to be accompanied by stimulus-dependent formation of DNA loops that bring in proximity enhancers with their target promoters (Schaukowitch et al., 2014; Joo et al., 2015). Activity-dependent DNA double-stranded breaks occur at the promoters of IEGs and are sufficient to drive their expression, possibly by facilitating the formations of such enhancer-promoter loops (Madabhushi et al., 2015).

Recent studies have also characterized the role of activity-dependent chromatin organization changes on a genome-wide scale (Dileep and Tsai, 2021). In response to neuronal stimulation, thousands of new genomic regions gain accessibility (Su et al., 2017). Activity-dependent enhancers are particularly enriched for binding sites for AP1, a dimeric transcription factor composed of Fos and Jun family proteins (Malik et al., 2014; Fernandez-Albert et al., 2019), which bind and open chromatin acting as pioneering factors (Vierbuchen et al., 2017). The enhancers gaining accessibility contribute to enhancer-promoter loops, which are thought to be important in initiating transcription (Fernandez-Albert et al., 2019). A recent study suggested that looping dynamics differs depending on ARG induction kinetics (Beagan et al., 2020). IEGs were found to form fewer, shorter loops, consistent with their fast induction and poised transcriptional state, whereas LRGs engage in both new and preexisting loops that are more complex and more slowly formed. Such changes in 3D chromatin conformation are being shown to be important players in adult neuronal plasticity (Yamada et al., 2019; Marco et al., 2020). Moreover, *de novo* formation of enhancer-promoter contacts was described during activity-dependent postnatal interneuron development (Stroud et al., 2020), whereas genome-wide changes in chromatin architecture during the critical period in V1 were shown to progress even without sensory inputs (Tan et al., 2021). Thus, it will be essential to discriminate and further characterize 3D chromatin reorganization mediated by hardwired genetic developmental and activity-dependent programs in future studies.

Overall, these results highlight that chromatin mechanisms are key regulators of activity-dependent transcription, ensuring the rapidity of the response, while also precisely regulating the developmental timing of IEG induction *in vivo.*

### Activity Pattern Specificity of Transcriptional Programs

Two key features that emerge from the work carried out in the experience-dependent transcription field are the cell-type and stimulation specificity of sensory programs (Yap and Greenberg, 2018; Gray and Spiegel, 2019; Tyssowski and Gray, 2019). The elucidation of these characteristics has been particularly facilitated by the advent of (single cell) genomic technologies which allow to probe the transcriptome and epigenome changes induced by different stimulations in distinct cell types or in single cells (Lacar et al., 2016; Hu et al., 2017; Wu et al., 2017). The specificity and heterogeneity of activity-dependent programs are key properties that are also likely to be useful to understand how spontaneous activity-regulated programs work during circuit assembly and will be discussed below.

The idea that differences in the type of neuronal stimulation lead to different transcriptional responses was initially shown by analyzing small subsets of known ARGs. Stimulating neurons with either growth factors or activity leads to the activation of different ARGs (Bartel et al., 1989). While CREB phosphorylation can be caused by many forms of stimulation, only those leading to calcium entry result in *Bdnf* transcription (West et al., 2002). The induction levels of Fos are dependent on the temporal pattern of the electrical stimulation both *in vitro* and *in vivo* (Sheng et al., 1993; Fields et al., 1997; Aydin-Abidin et al., 2008). These experiments already suggested that neurons can extract information about the type of the stimulus and about its distinct properties, and respond with a coherent transcriptional output (Lee and Fields, 2021).

Experiments taking advantage of genome-wide profiling have provided further evidence that the idea of transcriptional responses tuned to features of the stimulus does not only apply to selected genes, but to entire transcriptional programs, giving rise to the concept of stimulation-transcription map (Tyssowski and Gray, 2019). Chen and colleagues used transcriptional profiling in *Drosophila* following three protocols of neuronal stimulation and showed that the transcriptional responses are largely stimulation-specific (Chen et al., 2016). The transcriptional response to the same stimulation protocol was also different between distinct cell types. Using cultured spinal sensory neurons, Lee and colleagues delivered precise patterns of electrical stimulations that did not vary in the number of total action potentials or the duration of stimulation, but in the length of inter-burst periods (Lee et al., 2017). Transcriptional profiling showed that this subtle change can lead to the differential regulation of hundreds of transcripts, comprising both core IEG TFs as well as many other molecules with a variety of cellular functions, implicating neuronal firing kinetics in the activation of distinct gene regulatory networks (Lee et al., 2017; Iacobas et al., 2019).

The duration of activity is also a key feature of the stimulation (Lee et al., 2017; Tyssowski et al., 2018). By performing both brief and sustained stimulations *in vitro* and *in vivo*, it was shown that stimulation length is predictive of the transcriptional response. While sustained activity caused a transcriptional response consisting of three waves (rapid and delayed PRGs and secondary response genes) with distinct temporal kinetics, brief activity only induced rapid PRGs. These genes have permissive chromatin states at their regulatory elements prior to activation, and depend on the fast MAPK signaling pathway for their induction, properties that are unique compared to the two subsequent transcriptional waves (Tyssowski et al., 2018).

Comparing the transcriptomes resulting from decreased *vs.* increased neuronal activity showed that the two induced transcriptional programs are largely non-overlapping (Schaukowitch et al., 2017; Garay et al., 2020). The specific genes induced by these distinct programs have direct roles in the regulation of synaptic strength, so that shifts in activity can be compensated by synaptic upscaling or downscaling to reach homeostatic plasticity (Schaukowitch et al., 2017; Mao et al., 2018; Garay et al., 2020).

Excitatory post-synaptic inputs cause neuronal depolarizations, which in turn result in output action potentials. In hippocampal neurons, it was shown that the IEG TF NPAS4 is induced by distinct mechanisms: while action potentials cause *de novo* transcriptional induction, synaptic inputs enhance translation of *Npas4* mRNA localized in the proximal apical dendrites and its translocation to the nucleus. Furthermore NPAS4 forms stimulus-specific heterodimers that show distinct patterns of DNA binding and thus regulation of different target genes (Brigidi et al., 2019).

These results suggest that there are a number of cellular mechanisms that can communicate features of neuronal activity to the genome to mount a specific transcriptional response (Heinz and Bloodgood, 2020). For example, the temporal kinetics of the stimulus activate a distinct transcriptional response because a specific signaling pathway with similar kinetics is preferentially activated (Tyssowski et al., 2018; Lee and Fields, 2021). Differences in the dynamics of intracellular calcium oscillations have also been shown to selectively activate different sets of inducible TFs (Dolmetsch et al., 1997, 1998). Such constraints are not only found at the level of signaling pathways, but also in the epigenetic properties of the regulatory elements of ARGs, which make them poised for distinct temporal activation kinetics (Tullai et al., 2007; Tyssowski et al., 2018; Kitazawa et al., 2021). Enhancers might also contribute to the stimulus-specificity of the transcriptional response by combinatorially integrating the binding of TFs activated by distinct activity-dependent signaling pathways. Accordingly, *Fos* can be activated by different types of stimulations through its regulatory regions comprising five enhancers, which are engaged in a stimulus-specific combinatorial manner (Joo et al., 2015). Bdnf signaling-specific chromatin accessibility and TF binding of enhancers underlies the specificity of the transcriptional programs induced by Bdnf *vs.* neuronal depolarization (Ibarra et al., 2021).

### Cell Type Specificity of Transcriptional Programs

While neurons can decode different stimuli through their activity-dependent responses, the functional and molecular heterogeneity of neurons in the brain begs the question of whether different cell types show different transcriptional responses to a sensory stimulus, and whether these differences are relevant for neuronal plasticity. Studies of stimulus-dependent transcription in non-neuronal cell types suggest that while IEGs form a core set of stimulus-response genes in all systems, LRGs show context-specific expression and functional roles (Fowler et al., 2011; Yap and Greenberg, 2018). Transcriptional profiling of V1 at different timepoints of vision-dependent circuit maturation showed that IEGs are regulated by visual experience at all stages, while LRGs are largely induced in a stage-specific manner (Majdan and Shatz, 2006). This logic was then supported by comparisons of the activity-dependent transcriptional programs between classes (excitatory *vs.* inhibitory) or subclasses (different GABAergic cells) of neurons (Spiegel et al., 2014; Mardinly et al., 2016). Excitatory and inhibitory neurons *in vitro* and *in vivo* upregulate largely shared sets of IEGs, while LRGs are more neuron class-specific. The IEG *Npas4*, expressed by both neuronal classes, regulates transcriptional programs composed of both distinct and partly overlapping LRGs that are involved in modulating the number of inhibitory synapses on excitatory neurons and viceversa, thus controlling circuit homeostasis (Spiegel et al., 2014). Similarly, a specific subclass of cortical inhibitory neurons shows a distinct LRG program which controls its ability to disinhibit cortical circuits (Mardinly et al., 2016).

Hrvatin and colleagues carried out transcriptional profiling of thousands of single cells from visual cortex of dark-reared and light-stimulated adult mice and showed that both neuronal and non-neuronal cells exhibit robust experience-dependent transcriptional changes (Hrvatin et al., 2018). While IEG TFs were more likely to be shared across cell types and LRGs were more cell type-specific, the authors also found a considerable amount of specificity within the early response program. Only half of these TFs are expressed in more than three cell types. Accordingly, even though a set of IEGs is ubiquitous to both neuronal and non-neuronal types, another set is expressed in a mutually exclusive manner.

These results highlight the presence of both intrinsic and extrinsic influences that shape the specificity of activity-regulated programs. Differences in synapse to nucleus signaling have been reported between different neuronal classes (Cohen et al., 2016) and might account for differences at the level of the induced IEGs, but a key question is how this early response diverges to generate a more cell type-specific late response (Gray and Spiegel, 2019). Epigenetic mechanisms controlling the cell type specific state of activity-dependent enhancers (Malik et al., 2014; Spiegel et al., 2014), as well as their combinatorial activation by the cooperative binding of IEGs and cell identity-specific TFs (Andzelm et al., 2015; Vierbuchen et al., 2017; Yap and Greenberg, 2018), have been proposed to be a fundamental regulatory mechanism, but its relevance in the brain needs to be further determined.

Single-cell approaches have also been used in other sensory systems to highlight changes induced by perturbed auditory experience during critical periods for tonotopy in the auditory cortex (Barkat et al., 2020) and the effect of olfactory discrimination training *vs.* olfactory deprivation on the cell type composition of olfactory circuits (Tepe et al., 2018). Other studies, have highlighted that the combination of a cell connectivity pattern and activity state are key to drive its transcriptional identity, as it is the case for layer 6 cortical neurons with different connectivity patterns (Chevée et al., 2018), suggesting that sensory experience might fine-tune cellular differentiation in the late stages of development. Accordingly, in visual cortex, vision is required for the specification of intra-layer 2/3 cell subtypes and the dysregulation of an activity-dependent gene key for this process leads to disruptions in visual circuit function (Cheng et al., 2022). While the sensory experience-dependent programs regulating the refinement and plasticity of neuronal connectivity *in vivo* are emerging (Louros et al., 2014; Mardinly et al., 2016; Cheadle et al., 2018), those regulating the specification of cell identity remain to be characterized.

### Relevance for Spontaneous Activity Regulated Transcriptional Programs

**FIGURE 3:**
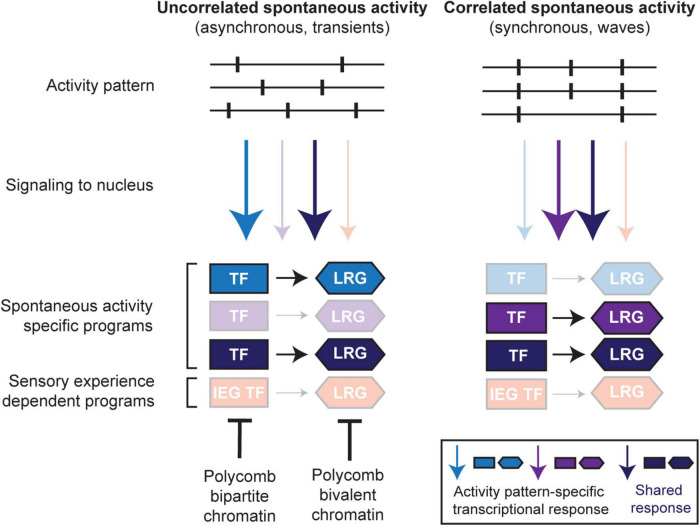
Spontaneous activity pattern-specific transcriptional programs. During neuronal development, spontaneous activity appears with various activity patterns (e.g., uncorrelated, left; correlated, right). Correlated spontaneous activity may itself appear with different patterns, e.g., synchronous bursting of neurons, different frequencies of bursts, waves of synchronous activity, etc. which might instruct specific signaling to the nucleus and transcriptional induction of spontaneous activity-specific transcription factors (TFs) and their downstream late response gene (LRG) effectors in a developmental stage- and/or cell type-specific manner. In addition, cell-intrinsic properties (e.g., cell membrane molecules, signaling pathways, chromatin organization) may also determine stage- and/or cell type-specific transcriptional responses to distinct neuronal activity. For example, at pre-sensory stages, precocious inappropriate activation of sensory experience-dependent IEG TF and LRG programs are suppressed by Polycomb-dependent bipartite and bivalent chromatin signatures, respectively (also see Figure 2).

The molecular mechanisms operating downstream of spontaneous activity are poorly understood. Nonetheless, a recurrent theme is that information encoded in the pattern of activity (e.g., the frequency of bursts) is key in instructing different developmental processes. In this optic, spontaneous activity may support circuit assembly through the implementation of transcriptional programs induced by its specific features (Figure 3).

There is a myriad of different patterns of spontaneous activity across sensory systems which dynamically change across the maturation of a given circuit (Figure 3, top panel; Maccione et al., 2014; Martini et al., 2021). In addition, developing neurons and circuits show distinct extrinsic and intrinsic properties, which are also dynamic during development. Extrinsic properties might correspond to transient synaptic and gap junction connectivity patterns, which influence the type of activity produced by a circuit (Figure 3, top panel; Blankenship and Feller, 2010; Colonnese and Phillips, 2018). The valence of synapses can also change during development; for instance, GABAergic neurotransmission in the early postnatal brain, until P9 in mice, is depolarizing and therefore important in driving spontaneous circuit activities, but subsequently shifts to become hyperpolarizing around the onset of sensory experience (Peerboom and Wierenga, 2021). Intrinsic properties can comprise distinct calcium signaling pathways (Figure 3, middle panel; Tyssowski and Gray, 2019), as well as different transcriptional and epigenetic states (Figure 3, bottom panel; Stroud et al., 2020; Kitazawa et al., 2021). This changing landscape of intrinsic and extrinsic features might thus specifically influence or constrain the genomic response to spontaneous activity (Figure 3, bottom panel).

Early studies in the spinal cord of developing *Xenopus* tadpoles showed that both calcium transients and calcium waves events occur with different frequencies (Gu and Spitzer, 1995). Blocking all calcium events interferes with neuronal differentiation, but neurite outgrowth and neurotransmitter specification can be restored independently by rescuing either waves or transients, respectively (Gu and Spitzer, 1995). During the pathfinding of TCAs, differences in the frequency of spontaneous bursts correlate with the expression of different effectors of axonal elongation speed (Mire et al., 2012; Castillo-Paterna et al., 2015). In *ex vivo* cortical cultures, *Bdnf* promoter activity is optimally induced only by certain patterns of stimulation (Miyasaka and Yamamoto, 2021). In the auditory system, two studies using single cell approaches have shown that the emergence of spiral ganglion neuron subtypes is an activity dependent process taking place before the onset of hearing (Shrestha et al., 2018; Sun et al., 2018). Crucially, genetic manipulations altering the frequency of cochlear spontaneous activity *in vivo*, without fully suppressing it, result in the failure to specify cellular subtypes, suggesting that the normal frequency of spontaneous activity might instruct this process (Sun et al., 2018). In the olfactory system, cell-autonomous differences in spontaneous activity patterns measured *in vivo* are instructive for the expression of a specific code of axon guidance molecules (Nakashima et al., 2019), but the transcriptional programs that connect the activity to the expression of specific effectors remains to be characterized.

The visual system might also represent a good entry point for experiments addressing spontaneous activity-regulated transcription due to the detailed characterization of the patterns of retinal waves, the presence of genetic tools to specifically alter them, and the knowledge of the resulting phenotypes (Leighton and Lohmann, 2016). These properties can be leveraged to uncover whether and how different features of spontaneous activity (e.g., wave frequency) engage different transcriptional programs. Importantly, the identity of the calcium-dependent signaling pathways, the inducible TFs, and the activity-dependent enhancers that they bind to drive expression of downstream effectors are key steps for a comprehensive understanding of the molecular mechanisms underlying retinotopy and eye-specific segregation.

## Conclusion and Outlook

In this review, we have highlighted that significant progress has been made in the fields of neuronal spontaneous activity and experience-dependent genome regulation. On the one hand, there is extensive work showing that before the onset of sensory experience, spontaneous activity of diverse origins, patterns, and functional roles is produced by the neurons of all sensory modalities and supports circuit assembly from axon guidance to refinement of synaptic connections and cell death. On the other hand, we have a sophisticated understanding of epigenetic chromatin and transcriptional regulation downstream of sensory experience, but current knowledge of how spontaneous activity communicates with the genome remains very limited. Most reports are focused on a handful of effector molecules, which are mainly studied in activity-dependent axon guidance. To our knowledge, no study has systematically probed the transcriptional programs downstream of spontaneous activity in sensory systems *in vivo.* The reasons for this gap in knowledge might include the difficulty in specifically manipulating spontaneous activity, in contrast to the relative ease of manipulating sensory inputs, which can be done with a variety of deprivation paradigms.

**FIGURE 4:**
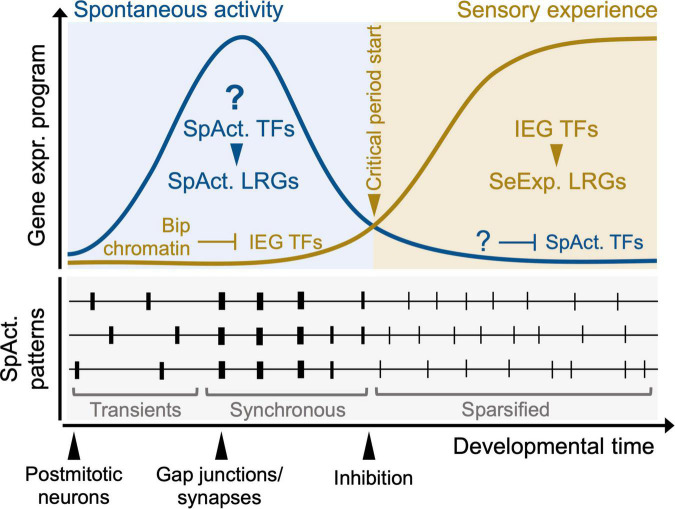
A model for the transition of activity-dependent transcriptional programs during sensory map development. Question marks indicate aspects that currently remain speculative. At pre-sensory stages, (top left, blue panel), the experience-dependent program (orange curve), composed of IEG TFs and sensory experience LRGs (SeExp. LRGs) is silenced by Polycomb-dependent bipartite (Bip) and bivalent (Biv) chromatin. At the onset of sensory experience (transition from blue to orange panel), Bip chromatin is resolved and IEGs and target LRGs are induced. During pre-sensory development, spontaneous activity (SpAct) patterns instruct initial sensory circuit wiring (top left, blue panel). A putative SpAct-dependent program (consisting of SpAct. TFs and target SpAct. LRGs, blue curve) is induced. At the onset of sensory-evoked activity (orange panel), the critical period plasticity starts and the SpAct-dependent program is downregulated. During the time-window of this transcriptional transition, spontaneous activity pattern change considerably (bottom panel). Post-mitotic neurons initially fire asynchronous transients and through synaptic connectivity and gap junction coupling their firing becomes synchronous. At the onset of sensory experience, spontaneous activity undergoes sparsification, resulting in a continuous decorrelated pattern. The circuit integration of inhibitory neurons is also thought to cause this transition in spontaneous activity. Whether these dynamic underlying activity patterns influence the proposed transcriptional changes remains to be addressed.

Another gap in our knowledge regards the potential interplay of spontaneous and sensory-evoked activities on the regulation of the canonical activity-dependent program (i.e., IEG TFs such as *Egr1, Fos, Jun*), which has been mainly studied after sensory stimulations (e.g., exposure to light or environmental enrichment), or pharmacological interventions (e.g., kainic acid) or *in vitro* (e.g., by KCl-mediated depolarization). In the spinal cord of *Xenopus*, early activity regulates neurotransmitter specification by phosphorylation of Jun, which in turn mediates the activity-dependent induction of *Tlx3* (Marek et al., 2010), suggesting that spontaneous activity can post-translationally activate IEG factors. Whether this applies to the developing sensory circuits in the mouse is less clear. In the somatosensory hindbrain, IEG expression is tightly controlled during embryonic development by the bipartite chromatin signature (Figures 2, 4, top panels; Kitazawa et al., 2021). *In vitro*, this form of epigenetic repression can be rapidly removed by KCl application, thus inducing IEGs in a matter of minutes. *In vivo*, the epigenetic repression of IEGs is relieved at perinatal stages (i.e., around E18.5). This corresponds to the earliest timepoint at which whisker stimulations can elicit cortical responses (Antón-bolaños et al., 2019), and marks the beginning of the critical period where inputs from the whiskers are required to refine the barrelette map (Erzurumlu and Gaspar, 2012). These results suggest that IEG induction follows the onset of sensory experience and that prenatal spontaneous activity in this pathway might thus rely on different transcriptional regulators (Figure 4, top panel). However, while there are *ex vivo* measurements of correlated activity in the embryonic hindbrain (Momose-Sato and Sato, 2013), and somatosensory thalamus, precise recordings in barrelette neurons have not been made.

These findings are also supported by a study of the transcriptional and epigenetic maturation of cortical interneurons, describing a switch in the chromatin and transcriptional program from the first to the third postnatal week, a period that corresponds to the onset of hearing, vision, and active whisking. In particular, while one week old interneurons express transcriptional regulators broadly associated with developmental functions (e.g., Ets, Neurod), the three week-old transcriptional program is dominated by activity-dependent factors, such as Fos, Jun and Npas4 (Stroud et al., 2020). The function of these TFs is also key for the activation of enhancers that are newly formed during this activity-dependent transition, while enhancers bound by developmental TFs get epigenetically repressed by DNA methylation, leading to the downregulation of the developmental program. In this optic, we speculate that a subset of the TFs in 1 week old neurons, which are downregulated at the onset of the IEG program, might be putative spontaneous activity-dependent effectors. In this hypothesis, TFs that are key for neuronal differentiation might be functionally co-opted as effectors of spontaneous activity. These questions need to be addressed by systematically investigating the developmental timing of IEG induction across sensory systems and comparing the transcriptional and epigenetic programs engaged during spontaneous activity- and experience-dependent circuit development.

While such transcriptional and epigenetic transitions around the onset of sensory activity are just starting to be elucidated, it is well appreciated that this time window sees important shifts in the physiological patterns of spontaneous activity in somatosensory and visual thalamocortical networks (Figure 4, bottom panel; Colonnese and Phillips, 2018; Nakazawa and Iwasato, 2021). Generally, neurons initially fire asynchronously (Figure 4, bottom panel, labeled “transients”), but later coupling by synapses and gap junctions leads to more synchronous firing events where local neurons show highly correlated activity (e.g., neurons within a cortical column are correlated; Figure 4, bottom panel, labeled “synchronous”). Right before sensory transduction, network activity shifts from correlated seldom events (neurons fire together periodically with long periods of silence in between) to decorrelated continuous events (Figure 4, bottom panel, labeled “sparsified”), which are similar to network activity during adult processing, which ensues at the start of sensory experience (Molnár et al., 2020; Nakazawa and Iwasato, 2021). This sparsification of cortical spontaneous activity has been proposed to rely on a reduced drive from the periphery (Gribizis et al., 2019; Nakazawa et al., 2020), formation of intra-cortical circuitry and the onset of functional inhibition in cortex (Romagnoni et al., 2020; Chini et al., 2021). The maturation of inhibition is also key for establishing the timing of cortical critical periods (Hensch, 2005), and it has been postulated that onset of the critical period might depend on inhibition preferentially targeting spontaneous *vs.* sensory inputs (Toyoizumi et al., 2013).

The developmental succession of these activity patterns, as well as the early evidence on the developmental timing of the canonical activity-dependent program onset, make it tempting to speculate that changing activity patterns promote different steps of sensory circuit formation by engaging distinct transcriptional programs (Figure 4). Assuming that the induction of IEGs is driven at the onset of sensory transduction (Figure 4, top right panel), the physiological properties of the activity at these stages might instruct the specific induction of this program. Similarly, this could explain why IEG transcription may not be induced by spontaneous activity (Figure 4, top left panel), although other mechanisms pertaining to the cellular state of developing neurons are likely also at play to prevent the inappropriate activation of different programs. Nevertheless, much about how spontaneous activity guides circuit assembly remains to be uncovered. Elucidating epigenetic chromatin regulation and the transcriptional programs downstream of spontaneous activity is a necessary endeavor towards this goal and will further our understanding of the stimulation-transcription coupling map during circuit development. Mutations in various components of the neuronal stimulation-transcription pathway as well as alterations in the normal patterns of spontaneous activity have been both found to be associated with neurodevelopmental disorders (Yap and Greenberg, 2018; Cheyne et al., 2019), highlighting how studies at the intersection of spontaneous activity and transcription might also hold relevance for disease.

## Author Contributions

GMP, TK, and FMR conceptualized the review. GMP wrote the first draft of the manuscript, which was revised by all authors. All authors contributed to the article and approved the submitted version.

## Conflict of Interest

The authors declare that the research was conducted in the absence of any commercial or financial relationships that could be construed as a potential conflict of interest.

## Publisher’s Note

All claims expressed in this article are solely those of the authors and do not necessarily represent those of their affiliated organizations, or those of the publisher, the editors and the reviewers. Any product that may be evaluated in this article, or claim that may be made by its manufacturer, is not guaranteed or endorsed by the publisher.
